# Supplementary material for: Association of Serum Proteases and Acute Phase Factors Levels with Survival Outcomes in Patients with Colorectal Cancer
Source: Cancers (Basel). 2024 Jul 6;16(13):2471. doi: 10.3390/cancers16132471 (PMC11240471; doi:10.3390/cancers16132471)
Supplement: Supplementary file 1 [file cancers-16-02471-s001.zip › cancers-3036238-supplementary.pdf]

SUPPLEMENTARY MATERIALS

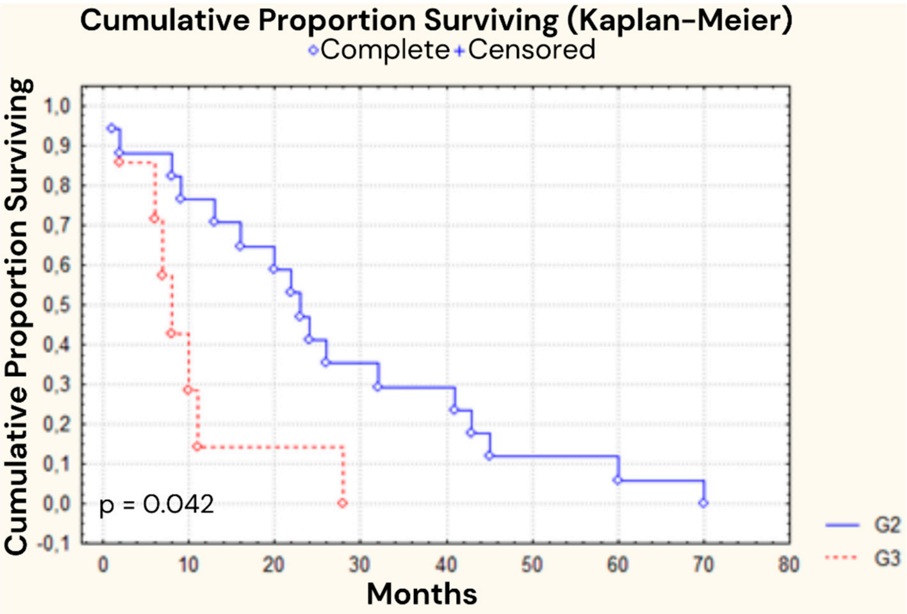

Figure S1. The survival analysis of patients with colon adenocarcinoma at various tumor grades. The Kaplan-Meier test was used to perform the survival analysis. p: statistical significance; G: grade.

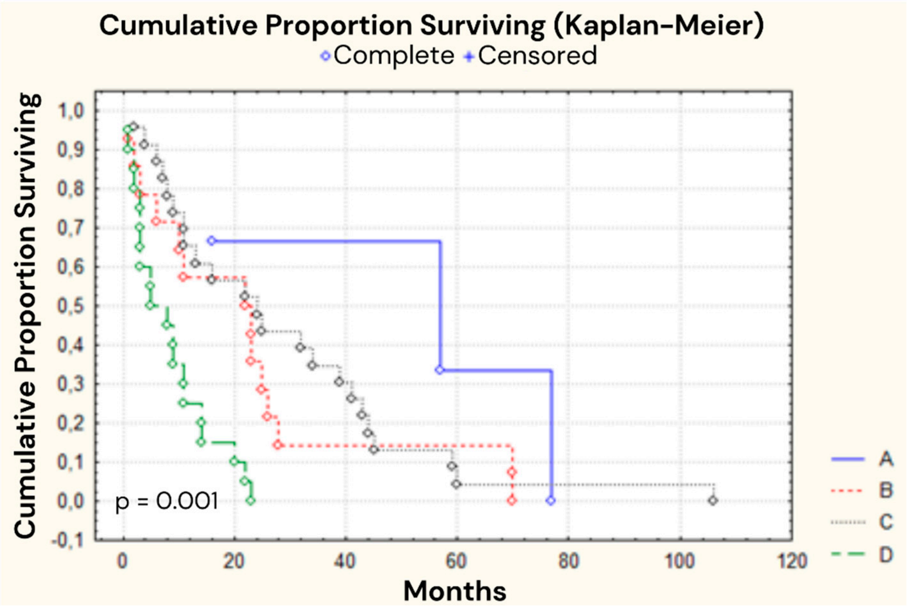

Figure S2. The survival analysis of patients with colon adenocarcinoma at various clinical stages. The Kaplan-Meier test was used to perform the survival analysis. Clinical stages (A, B, C, D) were assessed according to the Dukes classification. p: statistical significance.

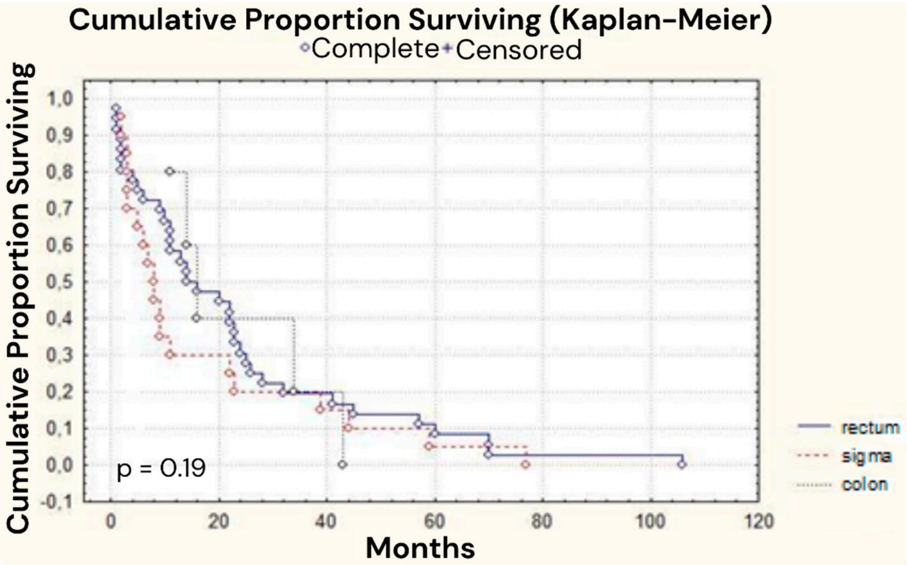

Figure S3. The survival analysis of patients with colon adenocarcinoma in different locations. The Kaplan-Meier test was used to perform the survival analysis. p: statistical significance.

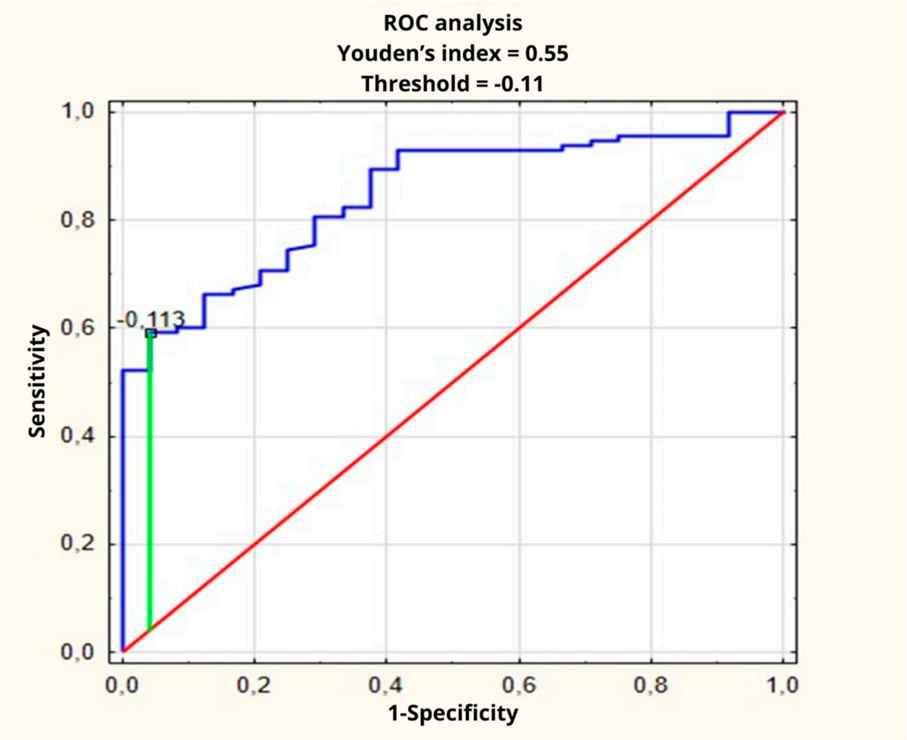

Figure S4. The receiver operating characteristic (ROC) curve used to determine the threshold for the combination of three parameters: leukocytic elastase, antitrypsin activity, and total sialic acid.
